# Photoactive Thin-Film Structures of Curcumin, TiO_2_ and ZnO

**DOI:** 10.3390/molecules26113214

**Published:** 2021-05-27

**Authors:** Anish Philip, Ramin Ghiyasi, Maarit Karppinen

**Affiliations:** Department of Chemistry and Materials Science, Aalto University, FI-00076 Espoo, Finland; anish.philip@aalto.fi (A.P.); ramin.ghiyasi@aalto.fi (R.G.)

**Keywords:** atomic/molecular layer deposition, spin coating, curcumin, titanium dioxide, zinc oxide, multi-layered structures, absorption

## Abstract

Curcumin is known as a biologically active compound and a possible antimicrobial agent. Here, we combine it with TiO_2_ and ZnO semiconductors, known for their photocatalytic properties, with an eye towards synergistic photo-harvesting and/or antimicrobial effects. We deposit different nanoscale multi-layer structures of curcumin, TiO_2_ and ZnO, by combining the solution-based spin-coating (S-C) technique and the gas-phase atomic layer deposition (ALD) and molecular layer deposition (MLD) thin-film techniques. As one of the highlights, we demonstrate for these multi-layer structures a red-shift in the absorbance maximum and an expansion of the absorbance edge as far as the longest visible wavelength region, which activates them for the visible light harvesting. The novel fabrication approaches introduced here should be compatible with, e.g., textile substrates, opening up new horizons for novel applications such as new types of protective masks with thin conformal antimicrobial coatings.

## 1. Introduction

Curcumin or diferuloyl methane (1,7-bis(4-hydroxy-3-methoxyphenol)-1,6-heptadiene-3,5-dione)—with the nickname “from-kitchen-to-clinic”—has been found potentially useful in pharmacology against various viral infections [1,2,3,4,5,6], besides its traditional use as a flavor in turmeric. The antiviral functionality of curcumin (Cur) is believed to stem from its ability to modify the protein structure of viruses, which can depress the activity of the virus or even prevent its entry to the target [1,7,8]. Recent reports have indicated that curcumin could be effective even against the coronavirus by inhibiting its encapsulation [8,9,10]; here, curcumin acts by suppressing the cytokine storm, thereby reducing the possibility of inflammation and multi-organ failure, i.e., the major reasons for the increased fatality of the coronavirus infection [8,10,11,12]. Additionally, curcumin has shown anticancer activity [2,13]. The active functional groups in the Cur molecule, i.e., the antioxidant hydroxyl group attached to a benzene ring and the bridging β-diketone group between two benzene rings, are significant for its antiviral property [10]. Moreover, the scavenging effect of curcumin on reactive oxygen species is considered important in its fight against various pathogens. Having the eye on its medical applications, it is important to supply curcumin in a way that maintains its active functional groups. Another issue is its poor bioavailability which makes oral treatments somewhat unfavorable [14]. Here, a possible solution could be to load, e.g., wearable masks or bandages with Cur molecules using a suitable thin-film coating technique to maintain the activity of curcumin.

In an early study [15], curcumin thin films were fabricated by simply immersing the substrate in a Cur-containing solution. However, this route involved amine compounds, which is a disadvantage considering the potential use of these coatings in healthcare. The conventional spin-coating (S-C) technique in which the thin film is grown by spinning the substrate with a solution containing the targeted molecules could be another facile route for the curcumin films. This technique has been extensively used for a wide variety of materials including metal oxides, polymers and even multilayered structures [16,17,18]. For bare curcumin films, little efforts have been made to exploit the S-C technique [15], but Cur-based metal complex films have been deposited using this technique [19,20]. In spin-coating, the film thickness can be controlled to some level by various experimental parameters (concentration and viscosity of the precursor solution, spinning time, rotation speed, temperature), but up-scaling and integration, e.g., with microelectronics are difficult. Additionally, solution routes are likely to leave some solvent inclusions in the growing thin film. Here, advanced gas-phase thin-film techniques, in particular the currently strongly emerging molecular layer deposition (MLD) technique could provide us intriguing options to address these challenges [21,22]. The MLD technique is an extension of the atomic layer deposition (ALD) technology originally developed for high-quality inorganic thin films needed, e.g., in microelectronics [23,24,25]. In MLD, the inorganic precursors are replaced by two mutually reactive gaseous/vaporized organic precursors to grow purely organic thin films of high level of controllability and quality. Similarly to ALD, the MLD technique has the capacity to yield precisely thickness-controlled, large-area uniform and conformal coatings on complex and/or sensitive surfaces such as biomaterials and textiles [26,27,28,29]. However, since the MLD technique is based on chemical gas-surface reactions between two different precursors with mutually reactive functional groups, e.g., hydroxyl and amine groups, it first of all requires a co-reactant (organic or inorganic), and secondly is likely to induce some changes in the primary precursor molecule when this molecule is reacting with the co-reactant to form new chemical bonds. Hence, in the case of the Cur-bearing thin films, the possible drawback could be that these changes would destroy the functional features of curcumin that are essential for its antiviral activity.

Recently we demonstrated the growth of titanium-curcumin (Ti-Cur) thin films using titanium (IV) isopropoxide (TTIP) as the co-reactant for curcumin [30]; note that in this case, the process is termed ALD/MLD as it involves both inorganic ALD (TTIP) and organic MLD (Cur) precursors [22,31,32]. It was shown that the Cur moieties are bonded to the Ti atoms via curcumin’s hydroxyl groups, but the β-diketone and methoxy groups were left intact. An attractive aspect in this ALD/MLD approach is that it is possible to further combine the ALD/MLD-grown metal-Cur layers with additional ALD-grown inorganic layers into different nanoscale multilayer structures [30,33,34]. In the present study, we will push this option forward, and also investigate the possibility to combine the ALD, MLD and S-C techniques to take a full advantage of their complementary benefits.

For the metal oxide components we have chosen ZnO and TiO_2_, as these two simple semiconducting oxides are not only non-toxic and biocompatible, but also known for their antimicrobial and photocatalytic properties [35,36,37]. Additionally, they are among the prototype materials in conventional ALD technology, hence well-behaving ALD precursors and processes are readily available for them [38,39]. The point of improvement with these oxides is the fact that the photocatalytic activity is limited to the UV range only, due to their wide bandgaps (ZnO: 3.3 eV, TiO_2_: 3.2 eV) [40,41]. Interestingly, there are few promising reports showing that curcumin may be used to sensitize/activate TiO_2_ towards longer wavelength (420–580 nm) absorption [42,43], or loaded in ZnO nanoparticles to enhance the antibacterial and anticancer properties [44]. Moreover, we have succeeded in tailoring the optical bandgap of TiO_2_ thin films by insertion of monomolecular organic (Cur or hydroquinone) layers into regular SL structures using ALD/MLD, such that the films were activated for visible-light absorption [30,45].

In this work, we demonstrate multiple novel ways of combining Cur molecules with both Ti and Zn metal cations and distinct TiO_2_ and ZnO layers into novel layer-engineered hybrid materials with the anticipation of intriguing synergistic effects. Most importantly, we fabricate well-defined superlattice (SL) structures in which monomolecular Cur layers are embedded within the TiO_2_ and ZnO matrices using the ALD/MLD technique, and also double-layer (DL) structures in which spin-coating is used to deposit nanoscale Cur layers which are combined with an ALD-grown TiO_2_ or ZnO layer. We address the benefits and challenges of each fabrication approach, regarding, e.g., the required deposition temperature and the resultant bonding schemes between the different layers. As one of the highlights, we demonstrate activation of these multilayer thin films for remarkable light absorption in the entire visible range, which could open up new horizons for a number of exciting applications.

## 2. Results

**Figure 1:**
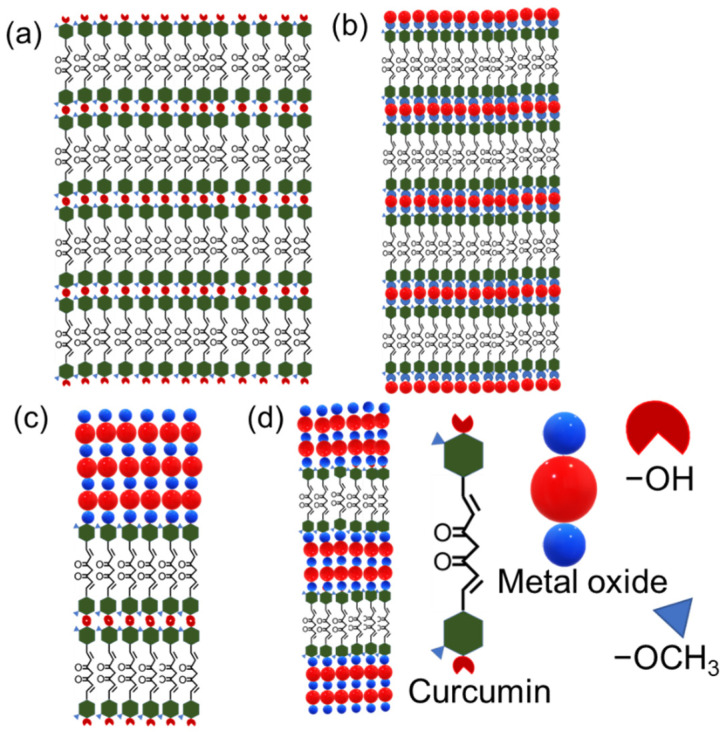
Thin-film structures deposited in this work: (**a**) Curcumin (Cur) films via S-C; (**b**) Metal-curcumin (Ti-Cur or Zn-Cur) films via ALD/MLD; (**c**) Curcumin plus metal oxide (Cur/ZnO or Cur/TiO_2_) double-layer (DL) films via S-C and ALD, respectively; (**d**) Metal-oxide:Cur (ZnO:Cur or TiO_2_:Cur) superlattice (SL) films via ALD/MLD.

By innovatively combining the S-C, ALD and MLD techniques, we were able to deposit various layer-engineered thin-film structures based on Cur, Zn-Cur, Ti-Cur, ZnO and TiO_2_ layers, as schematically illustrated in Figure 1. In the following sections, we first describe the new deposition processes developed for S-C-grown Cur films (Section 2.1), for ALD/MLD-grown Zn-Cur and Ti-Cur films (Section 2.2) and then for the different multi-layer structures (Section 2.3), and finally investigate the synergistic effects of the different layers on the UV-vis photoabsorption characteristics of these materials (Section 2.4).

### 2.1. Curcumin Films via Spin-Coating

**Figure 2:**
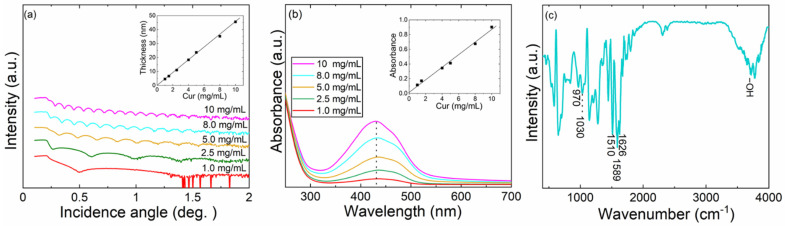
Characterization data for representative S-C-grown Cur films from Cur/ethanol solutions of different concentrations: (**a**) XRR patterns (inset: fitted film thickness versus Cur concentration); (**b**) UV-vis absorption spectra (inset: absorbance versus Cur concentration); (**c**) FTIR spectrum for the film grown from the 8 mg/mL solution.

With spin-coating we were able to readily deposit pure curcumin thin films in a highly reproducible manner. The thickness of the films could be controlled from 4 to 46 nm by increasing the Cur-solution concentration from 1 to 10 mg/m (the highest concentration limited by the solubility of Cur in ethanol). The XRR (X-ray reflectivity) patterns displayed in Figure 2a for the films reveal well-defined Kiessig fringes (oscillations) indicating the high quality of the films; from the continuously decreasing distance between these fringes with increasing starting-solution concentration, it can be seen that the resultant film thickness continuously increases. The inset in Figure 2a shows that this dependence of the film thickness on the Cur concentration in the solution is rather linear. The well-controlled S-C-growth of Cur films was further affirmed from the UV-vis absorption spectra (Figure 2b), showing the gradual (linear) increase of the absorption peak around 430 nm (originating from the π − π* electronic transition [46,47]) with increasing Cur concentration. From GIXRD (grazing-incidence X-ray diffraction) measurements, the films were found to be amorphous. The density of the films was estimated to be 2.12 g/cm^3^ from the XRR data.

Most importantly, the FTIR (Fourier transform infrared) spectrum shown for a representative sample in Figure 2c clearly reveals all the characteristic peaks due to the functional groups of curcumin [47,48]: presence of aromatic rings is seen from the peaks at 1589 cm^−1^ (symmetric aromatic ring stretching) and at 970 cm^−1^ (trans -C-H vibrations of aromatic ring), β-diketone groups from the peaks at 1510 cm^−^^1^ (C=O stretching) and at 1626 cm^−1^ (O=C-CH_2_-C=O moiety), methoxy groups (-OCH_3_) from the peak at 1030 cm^−1^ (C-O-C stretching vibration), and hydroxyl groups (-OH) from the large valley around 3500 cm^−1^. These features are essential for the antiviral functionality of curcumin [10].

### 2.2. Zn/Ti-Cur Films by ALD/MLD

**Figure 3:**
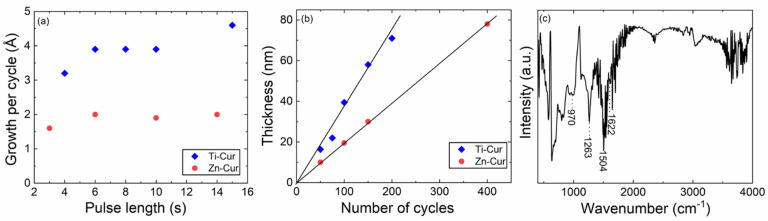
Characteristics of the ALD/MLD process of DEZ + Cur (in comparison to TTIP + Cur [30]) and the resultant Zn-Cur films: (**a**) Growth per cycle versus Cur pulse length; (**b**) Film thickness versus number of cycles; (**c**) FTIR spectrum for Zn-Cur film.

Through ALD/MLD, we were able to deposit metal-curcumin thin films with precise thickness control. In our previous work, we had already developed and optimized the process for the Ti-Cur films, based on TTIP (titanium isopropoxide) and Cur precursors [30]. The deposition temperature was 300 °C, defined by the relatively high evaporation temperature needed to vaporize curcumin (260 °C); the precursor/N_2_-purge pulsing sequence was optimized to be: 15-s TTIP/30-s N_2_/6-s Cur/15-s N_2_. In the present work, we carried out a similar process development/optimization for the Zn-Cur films at 300 °C; for the Zn precursor, the natural choice was diethyl zinc (DEZ) with the pulse/purge condition set to 2-s DEZ/3-s N_2_ based on extensive previous experience [38,49]. Then, we gradually varied the pulse length of Cur to find the length required for surface saturation; from Figure 3a, it can be seen that the saturation was reached with a 6-s Cur pulse. For the rest of our experiments with Zn-Cur films, we fixed the ALD/MLD precursor pulsing sequence as follows: 2-s DEZ/3-s N_2_/6-s Cur/15-s N_2_. This sequence yielded high-quality Zn-Cur films at 300 °C with a growth-per-cycle (GPC) value of 2.0 Å/cycle. In Figure 3b we compare the growth rates of the two processes, DEZ + Cur and TTIP + Cur, by plotting the film thicknesses as a function of the number of ALD/MLD cycles applied. For both the processes, this dependence is linear as expected, but the film growth rates are different. The lower growth rate (2.0 Å/cycle) for Zn-Cur compared to that for Ti-Cur (3.9 Å/cycle) is in line with the previous results for other ALD/MLD-grown Zn-organic and Ti-organic films [49,50].

In Figure 3c, an FTIR spectrum recorded for a representative Zn-Cur film (grown with 150 ALD/MLD cycles) is shown. Most significantly, the spectrum lacks the signature of the hydroxyl group stretching around 3500 cm^−1^ [48,51], indicating that the Cur molecules are bonded to the metal atoms via the hydroxy groups. On the other hand, the other features due to Cur are clearly visible in the spectrum, especially the peaks due to the aromatic rings at 970 cm^−1^ (trans -C-H vibrations of aromatic ring) and the β-diketone groups at 1504 cm^−1^ (C=O stretching vibration) and 1622 cm^−1^ (O=C-CH_2_-C=O moiety). This is important, as the deposition temperature (300 °C) was relatively high and could have led to decomposition of curcumin. The formation of the Zn-O bonds is seen from the sharp peak appearing at 1263 cm^−1^ (C-O stretching), in line with the Ti-O peak seen at 1261 cm^−1^ in Ti-Cur films [30]. From the GIXRD patterns (not shown here), both metal-Cur films were found amorphous, similarly to many other Zn-organic and Ti-organic thin films grown by ALD/MLD [49]. From the XRR data, the film density values were determined to be 2.32 g/cm^3^ for Zn-Cur and 2.16 g/cm^3^ for Ti-Cur, i.e., only slightly higher compared to value obtained for the S-C Cur films (2.12 g/cm^3^).

### 2.3. Superlattice and Double-Layer Structures with Curcumin and ZnO/TiO_2_

For the fabrication of the multi-layer superlattice and double-layer structures illustrated in Figure 1, we combined the S-C, ALD and ALD/MLD techniques. The S-C depositions were carried out at room temperature from a 8 mg/mL Cur/ethanol solution, while the ALD growths of the ZnO and TiO_2_ layers were performed at 120 °C, using the following pulsing sequences: 1-s DEZ/1.5-s N_2_/1.5-s H_2_O/2-s N_2_ for ZnO, and 6-s TTIP/15-s N_2_/3-s H_2_O/7-s N_2_ for TiO_2_ [30,49]. The ALD/MLD experiments for the ZnO:Cur and TiO_2_:Cur SL films were carried out at 300 °C using the deposition parameters presented in Section 2.2 for the DEZ/TTIP + Cur cycles, and repeating the precursor pulsing sequence: [(DEZ/TTIP + H_2_O)_m_ + (DEZ/TTIP + Cur)]_n_ + (DEZ/TTIP + H_2_O)_m_, where the chosen *n* value indicates the number of monomolecular Cur layers embedded within the metal-oxide matrix in the SL films.

**Figure 4:**
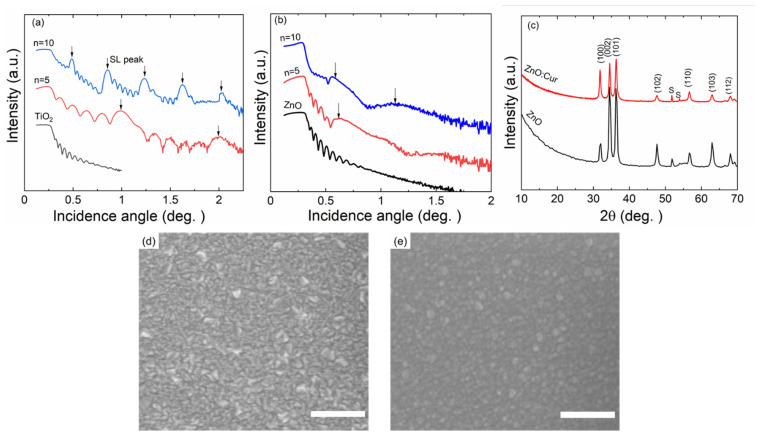
Characterization data for representative metal oxide:Cur SL films grown with ALD/MLD: (**a**) XRR patterns for TiO_2_:Cur films with different *n* values (=number of Cur layers) [30], SL peaks marked with arrows; (**b**) XRR patterns for ZnO:Cur films with different *n* values; (**c**) GIXRD patterns of ZnO and ZnO:Cur (*n* = 5) films, peaks from silicon substrate marked with s; top-view SEM images for (**d**) ZnO, and (**e**) ZnO:Cur films (scale bars are for 300 nm).

The intended multilayer (SL and DL) structures were verified with XRR. In Figure 4a,b we show XRR patterns for representative TiO_2_:Cur and ZnO:Cur superlattice films. For the former case, sharp and intense SL peaks are seen—as expected—between which the number of the smaller fringes increase with increasing *n* (=number of Cur layers). For the ZnO:Cur films, regular features are seen as well, but the SL peaks are less clear, indicating towards more diffuse interfaces between the ZnO and Cur layers (in comparison to those between the TiO_2_ and Cur layers). Another difference is that while the TiO_2_:Cur films are amorphous, the ZnO:Cur films are (poly)crystalline of the hexagonal wurtzite structure (representative GIXRD patterns are shown in Figure 4c). In previous works on ZnO:hydroquinone SL films, it has been shown that the ZnO grains may partly penetrate through monomolecular organic (hydroquinone) layers [52,53], which could be a plausible explanation here too for the somewhat smeared interfaces between ZnO and Cur. In Figure 4d,e, we display SEM (scanning electron microscopy) images for a representative ZnO:Cur SL film and a similarly grown (at 300 °C) bare ZnO film reference; it can be seen that the ZnO grains are of different shape in these two films, indicating some influence of the Cur layers on the ZnO grain growth. However, no significant differences in the average grain size are seen. The same conclusion applies to the average crystallite sizes calculated from the FWHM values of the most intense GIXRD peaks as follows: ZnO 14 nm, ZnO:Cur (*n* = 5) 18 nm and ZnO:Cur (*n* = 10) 12 nm. Finally, we like to mention that a common trend for ALD and MLD films is that amorphous films are smoother than the crystalline ones [54]. Hence, we expect that the interface roughness could be larger for the ZnO:Cur SL films, compared to the amorphous TiO_2_:Cur SL films. This could also contribute to the shallower XRR fringes for the ZnO:Cur SL films.

**Figure 5:**
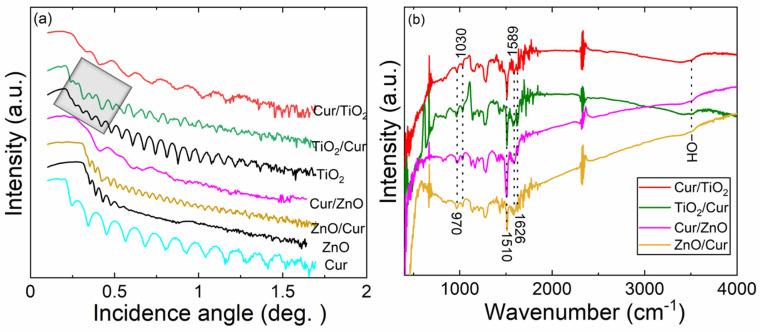
Characterization data for double-layer films: (**a**) XRR patterns, and (**b**) FTIR spectra (peaks due to Cur indicated) for the different metal oxide/Cur DL films, together with data for Cur, ZnO and TiO_2_ films for reference. An illustrative example of the change in the XRR fringe shape due to the DL structure is highlighted.

In Figure 5a, we display XRR patterns for different double-layer films, together with the Cur, ZnO and TiO_2_ films for reference. The first set of DL films, Cur/ZnO and Cur/TiO_2_, consist of a 35-nm thick S-C-grown Cur layer, and then a 10-nm thick ALD-grown metal oxide layer (ZnO or TiO_2_) on top of the Cur layer. The DL structure is visible for both of these films from the XRR patterns. The FTIR spectra (Figure 5b) for the same samples confirm that the curcumin is in principle well preserved upon the ALD growth of the metal oxide layer on top of it, judging from the FTIR bands seen for all the functional groups expected for curcumin. To address the impact of the order of the Cur and metal oxide layers in the DL films, we also fabricated TiO_2_/Cur and ZnO/Cur films for which the 35-nm thick Cur layer was spin-coated on top of a ca. 60-nm thick ALD-grown metal oxide layer. For these films, the FTIR and GIXRD data were essentially identical to the corresponding Cur/ZnO and Cur/TiO_2_ films. Nevertheless, the UV-vis absorption characteristics turned out to be somewhat different, as discussed in the following sub-chapter. Tentatively, we interpret this as an indication of diffusion of TTIP precursor molecules into the underlining Cur layer upon the ALD (TTIP + H_2_O) cycles. This phenomenon, called VPI (vapor phase infiltration) is well known for some ALD precursors when applied onto porous polymer substrates/surfaces [55].

Finally, regarding the crystallinity, we could conclude that all the aforementioned ZnO-based films were crystalline, whereas those based on TiO_2_ were amorphous. From existing ALD literature [30,39,45], it is known that the crystallinity of TiO_2_ films sensitively depends on the experimental parameters such as the deposition temperature and the film thickness. Hence, it was not unexpected to notice that not only the TiO_2_ layers deposited at 120 °C for the DL structures but also the SL films with ultrathin TiO_2_ layers between subsequent Cur layers (even though deposited at 300 °C) were amorphous. To demonstrate the impact of the deposition temperature, we carried out additional experiments to deposit the TiO_2_ layers for the TiO_2_/Cur DL films at 300 °C. Indeed, in this case the TiO_2_ layers were—similarly to bare 300 °C-deposited TiO_2_ films—(poly)crystalline (of the anatase structure). The crystallite sizes estimated from the GIXRD peak FWHM values were: TiO_2_ 33 nm and TiO_2_/Cur 30 nm.

### 2.4. UV-Vis Absorption Characteristics

**Figure 6:**
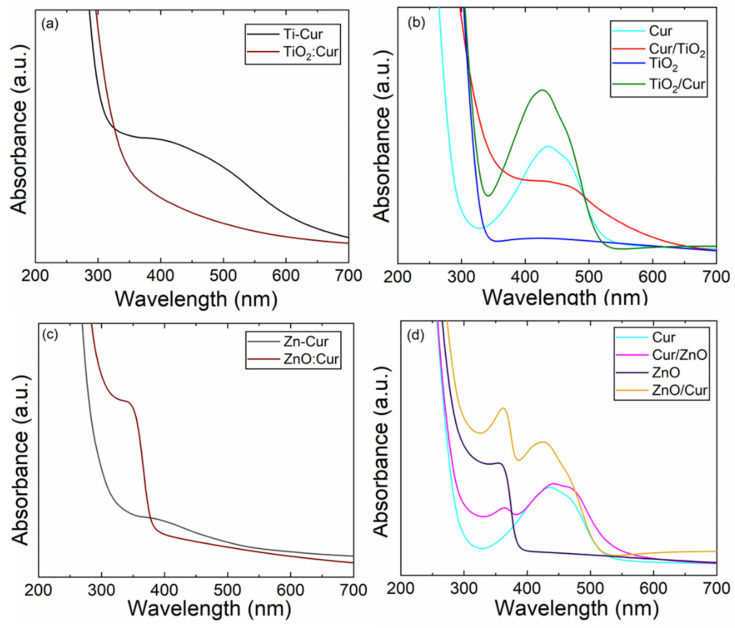
UV-vis absorption spectra for representative thin-film samples: (**a**) Ti-Cur and TiO_2_:Cur SL (*n* = 5), (**b**) Cur and TiO_2_, and their DL combinations, Cur/TiO_2_ and TiO_2_/Cur (**c**) Zn-Cur and ZnO:Cur SL (*n* = 5), (**d**) Cur and ZnO, and their DL combinations, Cur/ZnO and ZnO/Cur.

In Figure 6, we have collected representative UV-vis spectra for our thin-film samples from all the aforementioned sample categories. First of all, for the parent Cur, ZnO and TiO_2_ films, the absorption maxima (λ_max_) are seen—as expected—at 435 nm (due to the π − π* electronic transition of Cur [46,47]), at 360 nm [56], and at 422 nm, respectively. The slightly different λ_max_ value of 435 nm for our Cur film as compared to the previous data for curcumin solutions (ca. 430 nm) [57] may be due to the more closely packed Cur molecules in our S-C thin films. Then, the other broader absorption peak seen for Cur comprising the wavelength region 300~550 nm originates from its phenolic groups [46].

For the two ALD/MLD grown metal–Cur hybrid films, Zn-Cur and Ti-Cur, the λ_max_ is seen at 380 and 410 nm, respectively, indicating “curcumin-like” contribution even in these films where Cur is bonded to metal ions (via the OH groups). The absorption intensity is clearly lower for Zn-Cur in comparison to Ti-Cur [30]. The SL films, ZnO:Cur and TiO_2_:Cur, show similar features as the corresponding Zn-Cur and Ti-Cur films, but in a more diluted fashion (Figure 6a,c).

For the DL structures in which the ALD metal oxide layer was grown on top of the S-C Cur layer (Figure 6b,d), the absorption intensity of the characteristic Cur peak around 435 nm is decreased in intensity for Cur/TiO_2_, but is essentially intact for Cur/ZnO; in the latter case, instead, an additional peak appears at 365 nm due to ZnO which results in overall in a broad UV-vis absorption region (330~565 nm). Most interestingly, for Cur/TiO_2_ the 435 nm peak due to Cur strongly red-shifts, and as a consequence, the absorption edge of the Cur/TiO_2_ film extends far towards the visible end, up to ca. 650 nm. This feature looks similar to the absorption characteristics of the Ti-Cur films. Hence, we speculate that the possible penetration of TTIP precursor molecules into the underlining Cur layer upon the application of the ALD (TTIP + H_2_O) cycles on top of the spin-coated Cur layer could cause the formation of Ti-Cur bonds in the Cur/TiO_2_ DL films somewhat similar to those in the ALD/MLD grown Ti-Cur films. Note that we already shortly mentioned this possibility in Section 2.3. For the reverse-piled TiO_2_/Cur DL film, the penetration of the TTIP precursor molecules into the Cur layer naturally does not happen, and accordingly the UV-vis features are clearly different. Indeed, for the two reverse-piled DL films, TiO_2_/Cur and ZnO/Cur, it is seen that the Cur absorption feature persists and the 435-nm peak intensity increases from 0.34 for the bare Cur film, to 0.55 and 0.53 for TiO_2_/Cur and ZnO/Cur, respectively. Apparently, anchoring curcumin over the metal oxide layer (TiO_2_ or ZnO) enables additional photosensitization of Cur such that in overall enhanced absorption characteristics are seen for Cur-capped TiO_2_/Cur and ZnO/Cur DL films.

## 3. Materials and Methods

All the thin films were deposited on both 2.5 × 2.5 cm^2^ silicon and borosilicate glass substrates. For the spin-coating, the source solution was prepared by dissolving different amounts of curcumin (MERCK; curcumin for synthesis) in 99.5% ethanol (Altia) to vary the concentration of curcumin from 1.0 to 10 mg/mL. The spin-coating (WS-650SX-6NPP/LITE by Laurell Technologies) was carried out at room temperature by spinning the substrate at 2000 rpm for 2 min after dropping 0.25 mL of the Cur/ethanol solution.

For the ALD/MLD experiments, a commercial flow-type hot-wall ALD reactor (F-120 by ASM Ltd., Tsing Yi, Hong Kong) was used. The precursors were (sublimation temperatures in parentheses): curcumin (260 °C), deionized water (RT), titanium(IV) isopropoxide (25 °C) and diethyl zinc (RT). Both TTIP (Aldrich, 97%) and DEZ (Aldrich, 52 wt% Zn basis) were commercially purchased. It should be noted that we selected the less reactive TTIP instead of the more common TiCl_4_ as the precursor for titanium to avoid the unwanted side reactions that could arise from the interaction of TiCl_4_ with sensitive substrates, such textiles in the intended future applications. Two of the precursors, Cur and TTIP, were placed inside the reactor in open boats while the DEZ and deionized water cylinders were kept outside the reactor. Nitrogen (99.999%; flow rate at 300 SCCM) was used as both the carrier and purge gas. The reactor pressure was kept at 2–4 mbar. The ALD/MLD processes, DEZ/TTIP + Cur, were carried out at 300 °C, and the ALD processes, DEZ/TTIP + H_2_O, at 120 °C.

X-ray reflectivity (XRR; PANalytical X’Pert PRO Alfa 1; X’Pert Reflectivity software) were carried out for the verification of the multi-layer structures and for the determination of film thicknesses and densities. From the film thickness, the growth-per-cycle (GPC) value was calculated by dividing it by the number of deposition cycles applied. Film densities were calculated based on the critical angle θ_c_ dependency on mean electron density ρ_e_ of the film material, ρ_e_ = (θ_c_^2^π)/(λ^2^ r_e_), where r_e_ is the classical electron radius and λ is the X-ray wavelength [45]. The same diffractometer device was used to collect the grazing incidence X-ray diffraction (GIXRD) patterns for the films, with an incident angle of 0.5°. Crystallite sizes were calculated from the FWHM (full width at half maximum) values of the diffraction peaks using the Scherrer equation (Match software). The surface morphology and the grain sizes/shapes were studied with scanning electron microscopy (SEM; Hitachi S-4700). For the SEM analysis, the sample was mounted on a carbon tape and sputtered using an Au–Pd mixture. A current of 15 μA and voltage of 10 kV was used during the measurements.

For the identification of the different functional groups of the organic component and the metal-organic bonding features, Fourier transform infrared (FTIR, Bruker alpha II) spectroscopy was used. The interference from the silicon substrate was removed by subtracting the FTIR spectrum of the bare silicon substrate from the FTIR spectra of the samples. Finally, the UV−vis absorbance spectra (Shimadzu UV-2600 spectrometer) were recorded for the films deposited on glass plates in the wavelength range of 200–800 nm.

## 4. Conclusions

We have developed a set of approaches to deposit various curcumin-bearing thin films and multi-layer structures. Ultrathin curcumin films could be grown at room temperature in a highly reproducible way using the spin-coating technique. The valuable features of this process are: (i) the precise control on the films thickness (4~46 nm) by varying the concentration of curcumin solution, and (ii) the preservation of all the active functional groups of native curcumin, i.e., the β-diketone (O=C-CH_2_-C=O), methoxy (−OCH_3_) and the hydroxyl (−OH) groups. These are important considering the possible antiviral applications.

Another approach was to combine curcumin molecules with metal ions using the gas-phase ALD/MLD technique for the growth of metal-curcumin hybrid films; in these Zn-Cur and Ti-Cur thin films the curcumin moieties were found to bond to the metal atoms through their hydroxyl groups, but the β-diketone and methoxy groups were left intacted. The ALD/MLD technique allowed also to manipulate the metal-bearing and curcumin precursor supply sequences to grow superlattice structures in which the monomolecular curcumin layers are embedded within ZnO or TiO_2_ matrix. The UV-vis absorption characteristics observed for both the hybrid and superlattice thin films revealed promising synergistic effects beyond those seen for bare ZnO and TiO_2_.

Finally, we combined S-C-grown curcumin layers with ALD-grown ZnO or TiO_2_ layers for double-layer structures where the individual layers were of several tens of nanometers thick. Compared to the ALD/MLD approach, here the depositions could be carried out at significantly lower temperature (RT for S-C and 120 °C for ALD, while 300 °C for ALD/MLD), which is an advantage in case of temperature-sensitive substrates. Another advantage of the S-C plus ALD approach is the fact that this left the Cur layers intact so that we can expect them to show the antiviral functionality. The most interesting result was that even these separated layers mutually influenced the UV-vis absorption characteristics such that the absorption was not just a simple sum of the individual components. Most importantly, for the Cur/TiO_2_ DL film the absorption edge could be extended far towards the visible end, up to ca. 650 nm. This could be of considerable interest for the visible-light harvesting applications including photocatalysis. For Cur/ZnO DL film, a similar extension of the light absorption range to cover both UV and Vis ranges (330~565 nm) was seen, but the effect was less pronounced than in the case of Cur/TiO_2_. Finally, we observed that the order of the layer piling had a meaning as well. For example, in the case of Cur/TiO_2_ reversing the order of layers to TiO_2_/Cur enhanced the absorbance, indicating the positive effect of having curcumin as the top-layer.

## Data Availability

The data presented in this study are available on request from the corresponding author.

## Sample Availability

Samples of the compounds are not available from the authors.
